# The development of an online decision support tool for organizational readiness for change

**DOI:** 10.1186/1748-5908-9-56

**Published:** 2014-05-10

**Authors:** Sobia Khan, Caitlyn Timmings, Julia E Moore, Christine Marquez, Kasha Pyka, Galina Gheihman, Sharon E Straus

**Affiliations:** 1 Li Ka Shing Knowledge Institute, St. Michael’s Hospital, 30 Bond St, Toronto M5B 1W8, Canada; 2 University of Toronto, 563 Spadina Crescent, Toronto M5S 2J7, Canada

**Keywords:** Readiness for change, Readiness assessment, Decision support tool, Implementation, Implementation support, Implementation planning

## Abstract

**Background:**

Much importance has been placed on assessing readiness for change as one of the earliest steps of implementation, but measuring it can be a complex and daunting task. Organizations and individuals struggle with how to reliably and accurately measure readiness for change. Several measures have been developed to help organizations assess readiness, but these are often underused due to the difficulty of selecting the right measure. In response to this challenge, we will develop and test a prototype of a decision support tool that is designed to guide individuals interested in implementation in the selection of an appropriate readiness assessment measure for their setting.

**Methods:**

A multi-phase approach will be used to develop the decision support tool. First, we will identify key measures for assessing organizational readiness for change from a recently completed systematic review. Included measures will be those developed for healthcare settings (*e.g.*, acute care, public health, mental health) and that have been deemed valid and reliable. Second, study investigators and field experts will engage in a mapping exercise to categorize individual items of included measures according to key readiness constructs from an existing framework. Third, a stakeholder panel will be recruited and consulted to determine the feasibility and relevance of the selected measures using a modified Delphi process. Fourth, findings from the mapping exercise and stakeholder consultation will inform the development of a decision support tool that will guide users in appropriately selecting change readiness measures. Fifth, the tool will undergo usability testing.

**Discussion:**

Our proposed decision support tool will address current challenges in the field of organizational change readiness by aiding individuals in selecting a valid and reliable assessment measure that is relevant to user needs and practice settings. We anticipate that implementers and researchers who use our tool will be more likely to conduct readiness for change assessments in their settings when planning for implementation. This, in turn, may contribute to more successful implementation outcomes. We will test this tool in a future study to determine its efficacy and impact on implementation processes.

## Background

In recent years, healthcare systems have placed much importance on processes that motivate and enhance change within organizations, and that can ultimately lead to better implementation outcomes [1-3]. Measuring and establishing organizational readiness for change is one such process that can be performed in the initial phases of implementation [4-6]. Organizations increasingly recognize the benefits of performing readiness for change assessments to explore whether psychological and/or structural preparedness exists prior to implementation in their practice settings [7-11]; however, readiness assessments using valid and reliable measures are likely not being conducted [12]. Underutilization of valid and reliable organizational readiness measures may be the result of numerous challenges in the field [12,13], rendering it difficult to select an appropriate measure. Given the time and fiscal restraints that many organizations face, the process of selecting a valid and reliable assessment measure must be simplified to encourage the use of such measures in healthcare practice.

Acknowledging both the importance of readiness assessments and the underuse of assessment measures in practice, we seek to develop and test a rigorous decision support tool prototype that is designed to encourage more routine incorporation of a readiness assessment phase in implementation by facilitating the selection of appropriate readiness assessment measures.

### The importance of assessing readiness for change

Many healthcare organizations have prioritized the implementation of evidence-based innovations to improve the integration and cost effectiveness of services [7,8,14]. These innovations often necessitate some degree of behavioral change; for example, the implementation of a new electronic health record system that requires healthcare staff to learn new technological skills and transition their patient charting practices to suit a new electronic system [2]. Several factors affect whether this change in behavior will be successfully achieved at the individual and organizational levels—rendering implementation a complex and challenging process that often leads to little change, if any at all. As many as 60% to 80% of change strategies are not successfully implemented in healthcare [3].

Acknowledging that implementation is a time and resource-intensive process yielding sometimes unsuccessful results, implementation researchers have increasingly focused on examining the factors that contribute to effective implementation in practice settings. This research has revealed that one essential step of the implementation process is the assessment of organizational readiness for change (referred to interchangeably as ‘readiness’ or ‘readiness for change’)—defined as ‘the extent to which organizational members are both psychologically and behaviorally prepared to implement change’ [12,15,16]. Common implementation frameworks such as the Knowledge-to-Action (KTA) Cycle [4], Stages of Implementation [5], and Intervention Mapping [6] explicitly include steps involving readiness assessments. Furthermore, determining an organization’s level of readiness at the earliest stages of implementation (*i.e.*, exploration and planning) is associated with better implementation outcomes [1-3]. Recognizing the important role readiness plays in effective implementation, some funders and decision makers are now requiring organizations to assess and demonstrate readiness prior to implementing interventions [7-11].

Despite existing evidence on the importance of readiness, many organizational leaders do not accurately assess readiness for change prior to implementation [12]; for example, it has been estimated that half of unsuccessful implementation efforts are due to leaders overestimating their organization’s degree of readiness [12]. Recent systematic reviews [12,13,17] have identified substantial challenges in the field of readiness for change that can make it difficult to accurately assess readiness in practice; namely, discrepancies in operationalizing readiness for change, and navigating a large volume of published readiness for change measures in order to select the ‘right’ measure.

### Operationalizing readiness for change in organizations

One significant challenge in consideration of organizational readiness for change appears to be inconsistencies in a definition of readiness for change, whereby different assumptions are being made on what constitutes readiness for change at the organizational level [13]. In an effort to address these conceptual discrepancies, Weiner *et al.*[12] and Holt *et al.*[17] independently conceptualized readiness for change based on historical and theoretical definitions of readiness as existing at two levels—individual and organizational. Change at both levels is inextricably linked because the organizational level is a function of change at the level of the individuals that belong to the organization, but also at the level of the organization itself as a collective of individuals. Further refining this conceptualization, Holt *et al.*[17] suggested that readiness occurs both structurally, in terms of the circumstances and materials required to enact change, and psychologically, in terms of the capacity to process and accept change. According to Holt *et al.*[17], organizational change is defined by four key constructs that, taken together, constitute ‘readiness for change’:

1. Individual psychological (IP): Factors that reflect the extent to which individuals hold key beliefs regarding the potential change; recognize that a problem needs to be addressed; and agree with the changes required by individuals and the organization.

2. Individual structural (IS): Relevant dimensions related to the individual’s knowledge, skills, and ability to perform once the change is implemented.

3. Organizational psychological (OP): Relevant beliefs related to the organizational members’ collective commitment and collective efficacy.

4. Organizational structural (OS): Considerations related to human and material resources, communication channels, and formal policy.

These four constructs have been used to inform our understanding of organizational readiness for change.

### Measuring organizational readiness for change in practice settings

A second challenge in the field of organizational readiness for change is the large number and varying quality of existing organizational readiness measures. For example, in their review, Weiner *et al.*[12] identified over 40 available measures for organizational readiness for change. Very few of these measures (*i.e.*, 2% to 3%) have been assessed for validity or reliability. Moreover, it appears that many measures were developed for one-time use in specific projects and their psychometric properties have not been tested [12]; this latter testing is required to generalize the use of these measures to other settings. As a result, multiple measurement tools have been created for niche purposes. Still other measures largely duplicate existing measures with minor adjustments; the reasons for developing these measures rather than using established ones are unknown [12]. This phenomenon may relate to the varying conceptualizations of what constitutes readiness for change [13], and a perception that current tools do not meet the assessment needs of the organization.

In examining available measures, it is difficult to decipher what constructs of organizational readiness (*i.e.*, IP, IS, OP, OS) they are intended to assess. This leads to ambiguity regarding which measure(s) should be used to appropriately and accurately measure aspects of readiness for change that align with particular practice settings and assessment priorities. To complicate matters further, some existing measures assess concepts such as innovativeness or perceived need for change that are related to organizational readiness but do not accurately reflect readiness [12], rendering it difficult to reliably determine an organization’s true level of readiness.

To address the challenges discussed above and to encourage organizational readiness assessments in practice, we will develop and test a publicly available, online decision support tool to aid individuals in selecting an appropriate readiness for change measure for their setting. The purpose of the decision support tool is to guide users through the process of selecting valid and reliable readiness assessment measures given their organizational context, priorities for change, and unique implementation characteristics. This paper details the proposed methodology for developing and testing this decision support tool.

## Methods

To develop our decision support tool, we will use a four-phase approach. This will involve selecting valid and reliable readiness assessment measures; conducting a mapping exercise to assess the content of each measure; engaging a stakeholder panel to rate each of the selected measures; and creating and completing usability testing of an online tool for selecting an appropriate measure.

### Phase one: selection of valid and reliable readiness assessment measures

Our collaborators, affiliated with the Université Laval (Dr. Marie-Pierre Gagnon and Ms. Randa Attieh), recently conducted a systematic review of the frameworks and measures available to assess organizational readiness for change (Gagnon MP, Attieh R, Ghandour EK, *et al*. A systematic review of instruments to assess organizational readiness for knowledge translation in health care. Submitted for publication). The review was conducted using a standard systematic review methodology, including an established and piloted search strategy, abstract screening and data abstraction by two independent reviewers, and synthesis of results. The full protocol for the systematic review is published elsewhere [16]. The findings of this systematic review will be used to inform the development of our decision support tool as it is the most recent review of organizational readiness for change measures to date, and includes measures developed specifically for healthcare settings. The authors have also identified the validity and reliability for the various measures. Using the list of identified organizational readiness for change measures included in the review, we will identify valid and reliable measures that were developed for use in healthcare settings (*e.g.*, acute care, public health). We will not include measures designed to assess readiness for change in non-organizational settings (*e.g.*, community).

### Phase two: mapping items to a conceptual framework

Using measures that meet the inclusion criteria described above, we will conduct a mapping exercise to categorize what underlying factors/dimensions of organizational readiness each item included in each measure is intended to assess. The following information will be extracted independently from each measure by two investigators: setting of validation, innovation-specific focus (if applicable), and the inclusion of items assessing specific aspects of implementation beyond organizational readiness as defined by Holt *et al.*[17] (*e.g.*, developing an implementation plan). Inter-rater reliability between the two investigators will be computed using the kappa statistic; a score greater than 0.6 will be considered sufficient to proceed.

**Figure 1:**
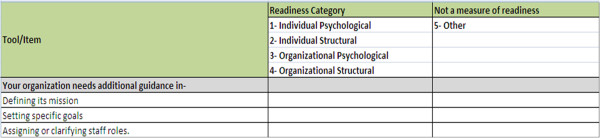
Mapping worksheet.

We will map each item of each of the included readiness assessment measures to one of the four readiness for change constructs as identified by Holt *et al.*[17], *i.e.*, individual psychological (IP), individual structural (IS), organizational psychological (OP), and organizational structural (OS), to determine which construct the item is most closely measuring, similar to mapping exercises conducted by Cane *et al.*[18] and Dixon *et al.*[19]. This conceptual framework was selected because it provides a multi-level depiction of organizational readiness that has informed definitions of readiness in the field [13,20]. Items that are inconsistent with any of the four constructs of organizational readiness will be categorized as ‘other.’ Before proceeding, the study investigators will reach consensus on the specific definitions of each of these constructs following from the work by Holt *et al.*[17] and Weiner *et al.*[12]. To facilitate the mapping exercise, we will create an electronic mapping worksheet (Figure 1). The worksheet will consist of the individual items abstracted from each of the selected measures; these items will be presented in random order, and reviewers will be blinded to the measure from which the items were derived.

A simple coding system will be developed for each of the four constructs of the organizational readiness for change framework (1 = IP; 2 = IS; 3 = OP; and 4 = OS). We will include an additional code for constructs lying outside of these definitions (5 = other), given that many measures contain items that do not assess readiness [12].

Four to six reviewers will independently review and code each item from each measure; as this methodology is similar to closed-sorting content validation tasks, two to 24 reviewers are sufficient for this exercise [19,21,22]. Reviewers to participate in the mapping exercise will be selected from among the study investigators as well as from external experts in the field. We will use the results of Attieh *et al.*’s systematic review [13] to purposively identify individuals who have published at least two articles on the topic of organizational readiness in healthcare (*i.e.*, ‘field experts’). Potentially eligible reviewers will be invited by e-mail to participate in the mapping exercise as an external reviewer, and will be given a brief overview of the study. Individuals who agree to participate will be sent an information package, instructions for completion, and a copy of the electronic mapping worksheet. Each reviewer (both internal and external) will conduct mapping independently over a two-week time period, and will send their completed worksheets by e-mail to a designated study investigator to collate the results.

To determine the level of agreement among the reviewers, data will be entered into SPSS 22.0 and an interclass coefficient (ICC) and 95% confidence interval (CI) will be computed. An overall ICC >0.70 will be considered satisfactory to move forward. Any discrepancies will be resolved through deliberation with the reviewers in consultation with all study investigators until final consensus is reached. Following deliberations, the proportion of items measuring each of the four constructs of organizational change readiness (*i.e.*, IP, IS, OP, or OS) and the ‘other’ category will be calculated per readiness for change assessment measure.

### Phase three: engagement of a stakeholder panel

After the completion of the mapping exercise, we will organize a stakeholder panel consisting of 20 to 25 individuals representing three participants groups: practitioners and managers from various types of healthcare organizations (*e.g.*, acute care, public health, long-term care); healthcare policymakers; and funders. To recruit stakeholders, we will use a purposive sampling approach. Recruitment will be supplemented by snowball sampling techniques as required. Potential participants will be invited to participate by e-mail.

Using a modified Delphi approach [23], individuals who agree to participate will be asked for feedback on the included readiness assessment measures in two rounds. In the first round, participants will be sent copies of the individual measures included in the mapping exercise by e-mail, and will be asked to complete an electronic rating form developed by the study investigators. They will be asked to rate the feasibility and relevance of each of the measures on a seven-point Likert scale. Study investigators will analyze the survey by computing median and response count on feasibility and relevance ratings. Content analysis will be conducted on open-ended comments. In round two, the results from round one will be disseminated to participants in a one-page summary distributed through e-mail. Participants will have an opportunity to review the findings and re-rate their responses or confirm their initial ratings. They will be asked to provide justification for changing their ratings (if applicable) in open-ended response boxes. Study investigators will use the same methods described above to analyze the results of round two.

Measures will be ranked by study investigators in order of most to least recommended based on average final stakeholder ratings; measures scoring highest on feasibility and relevance, in their respective categories, will be ranked as first or ‘most highly recommended’. These rankings will be provided to users of the decision support tool.

### Phase four: development and usability testing of an online decision support tool

In phase four, study investigators will use the results from the previous phases to develop a prototype of the readiness for change decision support tool. The mapping exercise findings will be the primary source of data used to create an algorithm for decision outcomes. To develop the algorithm, we will assume that the ideal readiness assessment measure can be selected by ranking the importance of each of the four constructs of organizational readiness [17] to the organization, in order of most to least important. Recommended measures should include higher proportions of items addressing readiness constructs that align with organizational priorities. Decision outcomes will also consider type of innovation and presence/absence of an established implementation plan. All possible combinations of decision outcomes will be considered. After the development of the algorithm, we will engage in: (i) the design of the decision support tool prototype, and (ii) usability testing of the prototype.

### (i) Design of the decision support tool prototype

The decision support tool prototype will be designed as an electronic survey to be completed by users. We anticipate that the prototype will likely include two sections. In the first section, users will be asked a series of questions designed to solicit information related to their implementation context. The second section of the tool will be used to identify the user’s priorities for readiness assessment. To inform section two of the prototype, we will develop a series of priority statements representing each of the four readiness constructs (IP, IS, OP, OS) (Table 1) that users will be asked to assess and rank from highest to lowest priority based on their knowledge of their organization.

**Table 1:** Readiness constructs and related statements for prioritization

**Readiness construct**	**Example statements**
Individual psychological	It is important to assess the beliefs, attitudes, and/or perceptions of individual staff members regarding the intervention.
Individual structural	It is important to assess the knowledge, skills, and/or abilities of individual staff members to deliver the intervention.
Organizational psychological	It is important to assess how effectively staff in the organization work together to achieve a common goal.
Organizational structural	It is important to assess the availability of human (e.g., staff, champions, leaders) and/or material (e.g., information technology, equipment, finances) resources to support the intervention.

Once the users complete the prioritization task, the tool will identify one or more recommended measures for their use. The results output will report on the feasibility and relevance of the measure(s) as rated by stakeholders, as well as the validity and reliability of the measure(s).

### (ii) Usability testing of the protoype

Usability testing will be completed to systematically evaluate the usability of the decision support tool prototype. The results of usability testing yield practical recommendations that can be applied to the structure, design, and redesign of the tool. Task-oriented measures such as navigation, successful use, and errors in use (*i.e.*, usability measures), as well as user experience indicators such as user preference, satisfaction, and understanding [24] will be determined. Usability testing will be done using expert, heuristic testing and individual usability testing. Heuristic testing, conducted by a human factors engineer, will be conducted to assess general usability of the tool, including examining the tool for consistency, organization, clarity of information, aesthetics, layout, legibility, and structure [25]. This process will lead to revision of the tool [24]. Following this, individual usability testing of the tool will be conducted.

In individual usability testing, individuals representing potential end users of the decision support tool (practitioners/managers, policymakers, and funders) will be recruited to participate in one-hour semi-structured interviews using a ‘think aloud’ methodology [26]. The goal of the individual sessions is to have participants use the tool and comment on any positive or negative aspects of usability measures or the user experience that may promote/hinder effective utilization of the tool. We will aim to recruit eight to ten individuals representing potential end users, which we anticipate will uncover over 85% of usability issues prevalent in the decision support tool [27]. Two cycles of individual usability testing may be required if major usability issues are identified; therefore, we anticipate recruiting a total of 16 to 20 participants in total. All usability testing sessions will be conducted online using WebEx live video conferencing, and will be audio recorded. Audio recordings will be transcribed verbatim, de-identified, and qualitatively analyzed by two analysts using a Framework Approach [28] with descriptive content analysis.

Following usability testing, the decision support tool will be revised and finalized. The end product of the readiness for change decision support tool will be hosted online, and will be freely and publicly available.

### Ethical approval

The proposed study has received approval from the Research Ethics Board of St. Michael’s Hospital in Toronto, Canada

## Discussion

The proposed study will result in the development of a decision support tool that will guide individuals interested in or preparing for implementation in the selection of a readiness for change assessment measure that is not only valid and reliable, but also appropriate for their needs and organizational setting. We will use rigorous methods of tool development to ensure that our readiness for change decision support tool can accurately select and recommend appropriate readiness assessment measures to a wide range of end users in the healthcare field. Furthermore, assessing the feasibility of our tool through usability testing will ensure that the final product will be easy to use and navigate, and will suit the needs of our target end users.

Given the findings of the recent systematic reviews on organizational readiness, it appears that readiness for change is still a field in its infancy [12,13,17]. There seems to be minimal understanding of how to effectively define and measure readiness, despite the importance of readiness for change in the implementation process having already been established [12,13,17]. However, while there is still work to be done to advance the field, a small number of valid measures exist [12], Gagnon MP, Attieh R, Ghandour EK, *et al*. A systematic review of instruments to assess organizational readiness for knowledge translation in health care. Submitted for publication.] that organizations can use to perform readiness for change assessments in their settings. Navigating the volume of literature to find these measures can be a daunting task for researchers and practitioners who are searching for an appropriate readiness assessment measure to utilize for their projects and contexts.

We hope that in developing this decision support tool, we will enhance knowledge on organizational readiness and bridge many important gaps that currently exist between research and practice. By addressing these challenges, we hope that our decision support tool will increase users’ confidence in using existing readiness for change measures, and increase their understanding of how and why these measures assess organizational readiness. After tool development and usability testing is completed, we plan to assess the impact of our decision support tool on enhancing readiness for change measurement by incorporating the tool into future implementation studies. Upon final release of the tool, we plan to make it available online for public use.

Once we have established inclusion criteria and processes for adding measures to the readiness for change decision support tool on an ongoing basis, we expect that it will be relatively simple to maintain the tool and to potentially expand the types of measures included in the tool to newly released/updated readiness measures in healthcare, as well as those validated for other settings and sectors. Ultimately, it is our aim for the products of this research to foster a shared understanding of organizational change readiness, and to encourage the use of readiness for change assessment in practice.

## Competing interests

Dr. Sharon E. Straus is an Associate Editor of *Implementation Science*. All decisions on this manuscript were made by another editor. The remaining authors have no conflicts of interest to declare.

## Authors’ contributions

SK and CT conceived of the study. SK, CT, JEM, CM, KP, GG, and SES participated in the design of the study and collectively drafted the research protocol. All authors read and approved the final manuscript.
